# Presentation and management outcomes of Retinoblastoma among Syrian refugees in Jordan

**DOI:** 10.3389/fonc.2022.1056963

**Published:** 2023-01-13

**Authors:** Yacoub A. Yousef, Qusai F. Abu Salim, Mona Mohammad, Imad Jaradat, Mustafa Mehyar, Reem AlJabari, Omar Al-Habahbeh, Khalid Saboubeh, Hadeel Halalsheh, Jakub Khzouz, Munir Shawagfeh, Iyad Sultan, Mahmoud AlMasri, Ibrahim Al-Nawaiseh, Maysa Al-Hussaini, Asem Mansour

**Affiliations:** ^1^ Department of Surgery, King Hussein Cancer Centre (KHCC), Amman, Jordan; ^2^ Department Radiation Oncology, King Hussein Cancer Centre (KHCC), Amman, Jordan; ^3^ Department Pediatrics Oncology, King Hussein Cancer Centre (KHCC), Amman, Jordan; ^4^ Department Pathology and Laboratory Medicine, King Hussein Cancer Centre (KHCC), Amman, Jordan; ^5^ Department Anesthesia, King Hussein Cancer Centre (KHCC), Amman, Jordan; ^6^ Department Diagnostic Radiology, King Hussein Cancer Centre (KHCC), Amman, Jordan

**Keywords:** enucleation, eye salvage, refugees, Retinoblastoma, survival

## Abstract

**Purpose:**

The humanitarian crisis in Syria has had a profound impact on the entire region. In this study, we report the patterns of presentation and management outcomes of Syrian patients with Retinoblastoma (Rb) treated at a single tertiary cancer center in Jordan.

**Methods and Materials:**

This is a retrospective comparative study of Syrian refugees and Jordanian citizens who had Rb between 2011 and 2020. Collected data included patient demographics, presentation, tumor stage, treatment modalities, eye salvage rate, metastasis, and mortality.

**Results:**

Thirty Syrian refugees (16 (53%) had bilateral disease) and 124 Jordanian citizens (51(41%) had bilateral disease) were diagnosed with Rb during this period. The median age at diagnosis for refugees was 10 and 32 months for patients with bilateral and unilateral Rb consecutively, compared to 6 and 28 months for citizens. The median lag time between signs of disease and initiation of treatment was 3 months for refugees, compared to 1 month for citizens.

Refugees were more likely to present with a more advanced stage (p=0.046). Out of 46 affected eyes in refugees; 32 (70%) eyes were group D or E, while out of 175 affected eyes among citizens; 98 (56%) eyes were group D or E. Therefore, refugees with Rb were more likely to mandate primary enucleation (48%) compared to citizens (25%) (p=0.003). However, out of 24 eyes among refugees who received conservative therapy, 15 (62%) eyes were successfully salvaged, while out of 131 affected eyes among citizens who received conservative therapy, 105 (80%) eyes were successfully salvaged (p=0.06). Two (7%) of the refugees and four (3.2%) of the citizens with Rb died from metastasis.

**Conclusion:**

Syrian refugees with Rb presented with more advanced disease due to delay in diagnosis and referral that increased the treatment burden by decreasing the chance for eye globe salvage. However, patients who received the timely intervention had a similar outcome to citizens with Rb; probably a reflection of the management of all patients at a single specialized center. We advocate for the timely referral of refugees with this rare life-threatening tumor to a specialized cancer center for the best possible outcome.

## 1 Introduction

Timely diagnosis and prompt management of Retinoblastoma (Rb), the most common primary intraocular malignancy in children linked to mutations in the *RB1* gene, are critical for the cure (1, 2). Globally, the incidence of RB is one in 15-20 thousand live births (3, 4). Although disease-specific mortality has markedly improved over the past years (5), global disparities in regional mortality rates remain a burning issue (6, 7). For example, the mortality rate in Asia and Africa, is as high as 40% to 70%, compared to less than 5.0% in Europe, Canada, and the United States (8–12). The incidence of Rb among the Jordanian population is estimated as one in 15620 newborns per year. Over the past few years, the mortality rate decreased from 38% to 5.0% because of the strict centralization of care for all Rb patients to a single specialized tertiary cancer center, in addition to the increased awareness among families and health caregivers in the country about this disease (13–16).

The humanitarian crisis in Syria, which started in 2011, has had a profound impact on the entire region. An estimated 5.6 million Syrians have fled their country, mainly to Turkey, Jordan, and Lebanon (17). Jordan hosts over 1 million Syrians; most of whom reside in host communities rather than in Refugee camps, where they have access to existing public health services (18). In 2018, cancer affected more than 18 million people worldwide, and more than 9.0 million died from the disease (19), and refugees are not immune (20, 21). Between 2011 and 2019, 917 Syrian cancer patients were registered at the King Hussein Cancer Center (KHCC) hospital-based cancer registry. A lack of sufficient funding from either host countries or international refugee aid organizations is expected to result in the suboptimal treatment of patients with cancer (22, 23).

Rb is a rare form of cancer, where a delayed diagnosis is considered the single most important poor prognostic factor in terms of survival and eye salvage rates (22–25). In a retrospective international study of 692 patients from 11 RB centers around the world, the long lag time between the first symptom for RB and visiting the RB treatment center was significantly associated with higher chances of an advanced tumor at presentation, presence of high-risk histopathology features, systemic metastasis and death (26). Refugees are challenged with difficulties in accessing adequate health care, which may result in delays in diagnosis and management of Rb, therefore are expected to have a worse outcome compared to citizens in the host countries. In this study, we evaluate the patterns of presentation and management outcomes among Syrian refugees living in Jordan, diagnosed with Rb who received treatment at King Hussein Cancer Center, in Amman, Jordan.

## 2 Materials and methods

This study was approved by the Institutional Review Board at King Hussein Cancer Center (IRB number = 22KHCC140). It was a retrospective, clinical case series of 124 Jordanian patients (citizens) and 30 Syrian patients (refugees) with 175 and 46 eyes affected with Rb, respectively, who had been managed at KHCC. Data from patients managed between 2011 and 2020 was analyzed. Inclusion criteria included Jordanian citizens and Syrian Refugee patients who had clinical and/or pathological diagnosis of Rb and were treated at KHCC and followed for at least 2 years after diagnosis. Syrian patients who are not refugees were excluded from this study.

Data collected included age at diagnosis, sex, laterality, affected site, International Intraocular Retinoblastoma Classification stage (IIRC) at diagnosis (26), presenting signs and symptoms, the lag time between the presenting sign and starting treatment, modality of treatment, eye salvage, metastasis, and mortality. Selection and data collection required access to patients’ medical records and Ret-Cam images.

### 2.1 Treatment modalities

We used a combination chemotherapy regimen of CVE (carboplatin, vincristine, and etoposide). Each CVE cycle was repeated every 4 weeks for a total of 6-8 cycles according to the patient’s condition and tumor status. Ocular oncology follow-up was provided with examination under anesthesia before every cycle of chemotherapy and every 4 weeks thereafter. Fundus photos were taken using a RetCam II (Clarity Medical System, Pleasanton, CA, USA). Combination focal therapy was applied as needed as Trans pupillary thermotherapy (TTT) and/or triple freeze-thaw cryotherapy (MIRA CR 4000). External beam radiation therapy was administered when needed consistently by applying 45 Gy in 25 fractions.

### 2.2 Statistical analysis

Descriptive analysis was carried out using mean, median, and range. Comparative analysis was carried out between refugees and citizens, and the P value was measured using Fisher’s exact test to analyze each factor’s predictive power.

## 3 Results

Over 10 years (2011-2020), 124 citizens and 30 refugees met our inclusion criteria.

### 3.1 Refugees Rb patients

**Figure 1:**
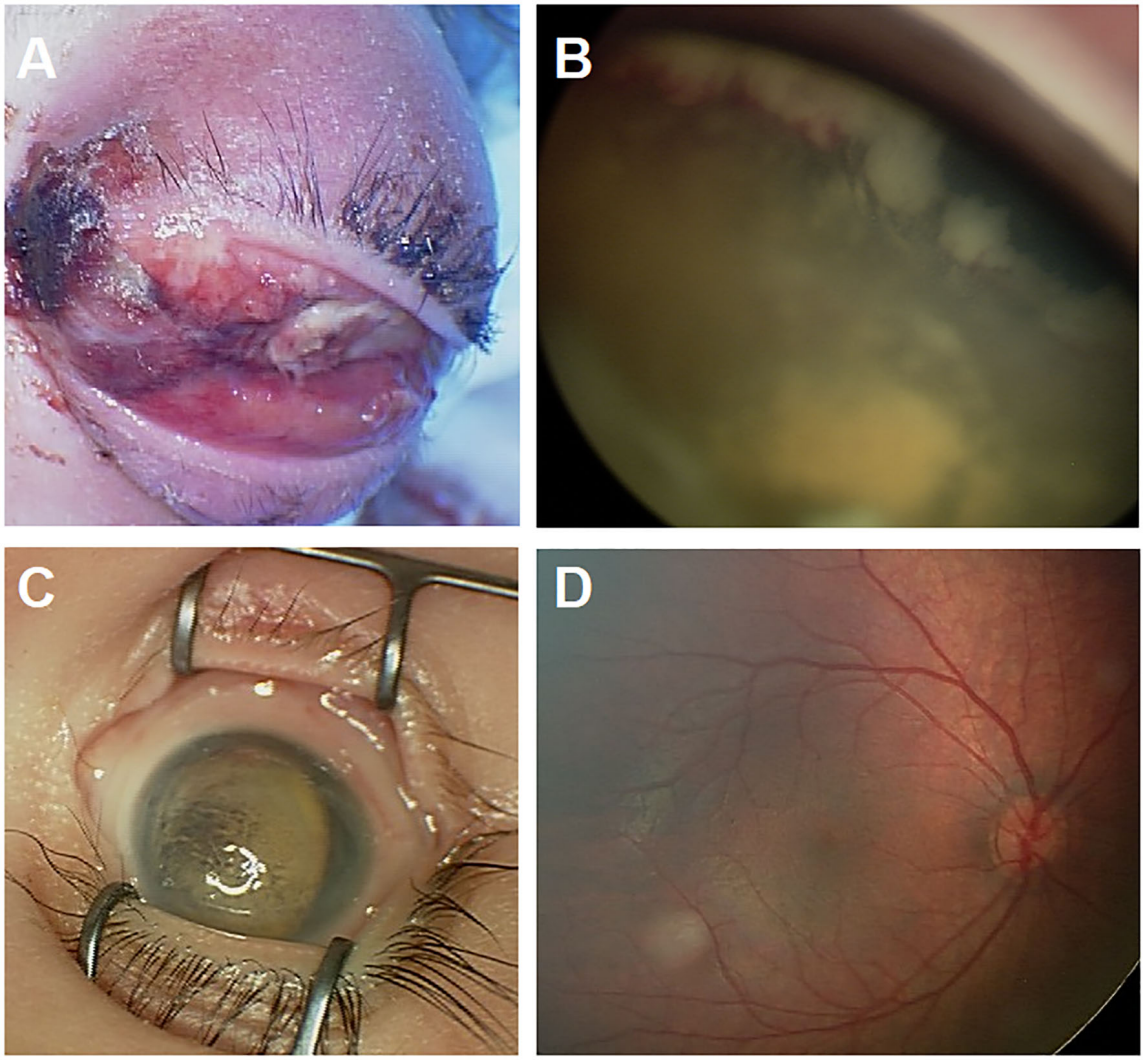
**(A)** Represents one of the refugees who came after 6 months of having leukocoria as a sign of ocular disease in the left eye and did not seek care until the patient developed extraocular disease extension. She passed away with bone marrow metastasis. **(B, C)** This patient is a refugee who presented with bilateral advanced intraocular Retinoblastoma. The right eye **(B)** showed extensive tumor that is extending to the ciliary body, and the left eye **(C)** had phthisis bulbi due to advanced disease, and unfortunately ended with bilateral enucleation. **(D)** This child was diagnosed by screening and had right group A tumor.

Out of 30 refugees with Rb, 12 (40%) were males, and 18 (60%) were females. Sixteen (53%) patients had bilateral Rb, and 14 (47%) had unilateral Rb. The median age at diagnosis was 10 months for patients with bilateral disease and 32 months for patients with unilateral disease. The median lag time between signs of disease and starting treatment was 3 months. The most common presenting sign was leukocoria in 16 (53%) patients, followed by strabismus in 6 (20%) patients, poor vision in 3 (10%), buphthalmos in 3 (10%) patients, 1 (3%) patient was diagnosed by screening (based on family history of Rb), and 1 patient presented with extraocular disease. Out of 46 affected eyes, 13(28%) eyes were collectively IIRC group A, B, or C, 32 (70%) eyes were group D or E, and 1 (2%) had an extraocular disease at the time of diagnosis (**Figure 1** and **Table 1**).

**Table 1:** Demographics, Tumor features, and management outcomes for 30 Syrian refuges with retinoblastoma treated between 2011-2020.

			Number	%	
Median age at diagnosis (Bilateral, Unilateral)			10 months, 32 months		
Gender		Male	12	40%	
		Female	18	60%	
Laterality		Unilateral	14	47%	
		Bilateral	16	53%	
Side		Right	10	33%	
		Left	4	13%	
		Both	16	54%	
Familial RB					
Presenting signs		Leukocoria	16	53%	
		Squint	6	20%	
		Poor vision	3	10%	
		Buphthalmos	3	10%	
		By screening	1	3%	
		Extraocular disease	1	3%	
The median lag time between signs of the disease and starting treatment			3 months		
	Number (%)	1ry enucleation (%)	Conservative treatment	Salvage for amended treatment.	Overall Eye Salvage
Number of eyes	46	22 (48%)	24 (48%)	15 (62%)	15 (32%)
IIRC stage					
A	4 (9%)	0 (0%)	4 (17%)	4 (100%)	4(100%)
B	3 (7%)	0 (0%)	3 (12.5%)	3 (100%)	3(100%)
C	6 (13%)	0 (0%)	6 (25%)	5 (80%)	5 (80%)
D	17 (37%)	8 (36%)	9 (37%)	4 (44%)	4 (24%)
E	15 (33%)	13 (59%)	2 (8%)	0 (0%)	0 (0%)
Extraocular*	1 (2%)	1 (100%)	0 (0%)	0 (0%)	0 (0%)
Metastasis	2 (7%)				
Secondary malignancy	0 (0%)				
Mortality	2 (7%)				

*This eye with the extraocular disease was treated by enucleation after neoadjuvant chemotherapy.

Treatment and outcome: Twenty-two (48%) eyes were treated by primary enucleation. In comparison, 24 (52%) eyes received conservative therapy (combined systemic chemotherapy and focal consolidation therapy), and 15 (32%) eyes in this group were successfully salvaged by the last date of follow-up, while secondary enucleation was the outcome for 9 eyes (overall, enucleation was the treatment for 31 (67%) of the total affected eyes). Two patients (7%) had metastasis and died by the last follow-up date (**Table 1**).

### 3.2 Citizens’ Rb patients

Out of 124 affected patients, 69 (56%) patients were males and 55 (44%) patients were females. Fifty-one (41%) patients had bilateral disease, and the rest had unilateral disease. The median age at diagnosis was 6 months for patients with bilateral disease and 28 months for patients with unilateral disease, and the median lag time between signs of disease and starting treatment was 1 month. The most common presenting sign was leukocoria in 88 (77%) patients, followed by strabismus in 25 (20%) patients, poor vision in 5 (4%), buphthalmos in one (1%) patient, and five (4%) patients were diagnosed by screening (based on family history of Rb). Out of 175 affected eyes, 77 (44%) eyes were IIRC group A, B, or C, collectively, while 98 (56%) eyes were group D or E at the time of diagnosis (**Table 2**). None of the patients had an extraocular disease.

**Table 2:** Demographics, Tumor features, and management outcomes for 124 Jordanian patients with retinoblastoma treated between 2011-2020.

			Number	%	
Median age at diagnosis (Bilateral, Unilateral)			6 months, 28 months		
Gender	Male		69	56%	
	Female		55	44%	
Laterality	Unilateral		73	59%	
	Bilateral		51	41%	
Side	Right		35	28%	
	Left		38	31%	
	Both		51	41%	
Familial RB			18	15%	
Presenting signs	Leukocoria		88	71%	
	Squint		25	20%	
	Poor vision		5	4%	
	buphthalmos		1	1%	
	By screening		5	4%	
	Extraocular disease		0	0%	
The median lag time between signs of the disease and starting treatment			1 month		
	Number (%)	1ry enucleation (%)	Conservative treatment	Salvage for amended treatment.	Overall Eye Salvage
Number of eyes	175	44 (25%)	131	105 (80%)	105 (60%)
IIRC stage					
A	12 (7%)	0 (0%)	12	12 (100%)	12 (100%)
B	24 (14%)	0 (0%)	24	23 (96%)	23 (96%)
C	41 (23%)	0 (0%)	41	38 (93%)	38 (93%)
D	75 (43%)	22 (29%)	53	32 (60%)	32 (43%)
E	23 (13%)	22 (96%)	1	0 (0%)	0 (0%)
Extraocular	0 (0%)				
Metastasis	4 (3.2%)				
Secondary malignancy	1 (1%)				
Mortality	4 (3.2%)				

Treatment and outcome: Forty-four (25%) eyes were treated by primary enucleation, while 131 (75%) eyes received conservative therapy (combined systemic chemotherapy and focal consolidation therapy), and 105 (60%) eyes in this group were successfully salvaged by the last date of follow-up while secondary enucleation was the outcome for 26 eyes (overall, enucleation was the treatment for 70 (40%) of the total affected eyes). Four patients (3.2%) had metastasis and died by the last date of follow-up (**Table 2**).

### 3.3 Comparison between citizen and refugees Rb patients

There was no difference between citizens and refugees in terms of sex, laterality, and incidence of familial diseases. The median age at diagnosis for citizens with Rb was 6 and 28 months for patients with bilateral diseases and unilateral disease, consecutively, while it was 10 and 32 months for refugees (**Table 3**). Although there was no statistically significant difference between both groups in terms of presenting symptoms, the refugees showed a tendency to more advanced signs as 10% of refugees with Rb had buphthalmos (compared to 1% of citizens) and 3% presented with an extraocular disease compared none the citizens (**Table 3**), In addition, only one single case was diagnosed by screening in Refugees versus 5 cases in citizens. The median lag time between signs of disease and starting treatment was 1 month for citizens and 3 months for refugees with Rb.

**Table 3:** Comparison between Jordanian RB patients and Refuges with RB diagnosed and treated at King Hussein Cancer Center 2011-2020.

		Citizens		Refugees		P-value
		Number	%	Number	%	
Number of patients		124		30		
Number of eyes		175		46		
Median age at diagnosis (Bilateral, Unilateral)		6 & 28 months		10 & 32 months		
Gender	Male	69	56%	12	40%	0.133
	Female	55	44%	18	60%
Laterality	Unilateral	73	59%	14	47%	0.159
	Bilateral	51	41%	16	53%
Familial RB		18	15%	6	20%	
Presenting signs	Leukocoria	88	71%	15	50%	0.217
	Squint	25	20%	5	17%
	Poor vision	5	4%	3	10%
	Buphthalmous	1	1%	3	10%
	By screening	5	4%	3	10%
	Extraocular disease	0	0%	1	3%
The median lag time between signs of the disease and starting treatment		1 month		3 months		
IIRC stage						
A, B, C		77	44%	13		0.046
D, E		98	56%	32	
Extraocular		0	0%	1	
Enucleation as primary treatment		44	25%	22	48%	0.003
Overall eye globe salvage rate		105	60%	15	32%	0.0014
Eye globe salvage rate for amended treatment		105	80%	15	62%	0.06
Metastasis		4	3.2%	2	7%	0.33
Mortality		4	3.2%	2	7%	0.33

Refugees presented with a more advanced stage than citizens, as 70% of eyes in refugees with Rb were classified as group D or E eyes at diagnosis compared to 56% of the citizens (p= 0.046). Therefore, refugees mandated primary enucleation more than citizens did (p=0.003). Even though the overall eye salvage rate for citizens was higher than for refugees (60% compared to 32%, P =0.0014), the eye salvage rate for eyes that received conservative therapy was comparable (80% versus 62%, p= 0.06). Finally, there was no statistically significant difference in metastasis and overall survival rates (**Table 3**).

## 4 Discussion

Cancer care and care for rare diseases, for both adults and pediatrics, might not be a priority of refugees’ health care in many host countries. Financial support is typically directed toward housing, food, and basic life needs. Ethical dilemmas ensue in such circumstances, especially when dealing with potentially curable cancers at a reasonable cost. Retinoblastoma is a rare life-threatening cancer in children that is curable if diagnosed early and treated adequately (4, 6, 10, 13). In this study we found, on average, a 2 months difference in lag time between groups in the diagnosis and initiation of treatment. This delay reduced the chance for eye globe salvage, without a significant impact on mortality.

The age-standardized rates (ASR) for age groups (0-14 and 0-19) for Rb were variable between different countries in the MENA region. The highest ASRs and the second highest worldwide were in Morocco with 9.2 and 7.1 cases per million person-years for the 0-14 and 0-19 age groups respectively. Followed by second place in Jordan with 7.1 and 5.1 cases per million person-years in both groups respectively. While the lowest ASRs recorded were in Qatar with 0.4 and 0.3 cases per million person-years years for the 0-14 and 0-19 age groups respectively. Tunisia recorded the same ASRs as Lebanon, with 3.6 and 2.8 cases per million person-years for the 0-14 and 0-19 age groups, respectively (27). Rb patients with delayed diagnosis and referral are expected to present with a more advanced tumor stage (25, 26, 28–30). In this study, 70% of the affected eyes in refugees had advanced intraocular stage (staged as group D or E), and 2% had an extraocular disease at diagnosis while only 56% of the affected eyes in Jordanian patients belonged to both groups, and none had an extraocular disease at diagnosis. This difference is expected because of the difference in the age at diagnosis directly related to the time lag between the first sign of the disease and the time of starting treatment. Our results showed that the median age at diagnosis for citizens with Rb was 4 months younger than the median age at diagnosis for refugees for both bilateral and unilateral patients. Furthermore, an extra 2-month lag time between signs of disease and starting treatment among refugees was noted in comparison to citizens.

The silent nature of Rb in the early intraocular stages, and the difficulties most refugee patients worldwide encounter in accessing medical care, may contribute to the late presentation seen among the patients in our study. Furthermore, the increased awareness of the disease, easy accessibility to health care, and the presence of the screening program for Rb in Jordan contributed to the difference in the promptness of diagnosis and starting treatment among Jordanian patients (29). For example, five Jordanian patients in this series were diagnosed by screening, compared to only a single refugee patient who had a parent and sister diagnosed with bilateral Rb. The signs and symptoms of Rb depend on its size and location. Leukocoria is the most frequent presenting sign of Rb, reported in approximately 50–60% of cases, followed by strabismus (25%) and inflammatory signs (6–10%) (30–33). In our study, 53% of refugees patients presented with leukocoria, followed by strabismus (20%) and buphthalmos (10%), in comparison to 71% with leukocoria, 20% with a squint and 1% with buphthalmos among Jordanians.

Unfortunately, the impact of the delay in diagnosis was reflected in the treatment burden and management outcomes. Rb management guidelines recommend that the early diagnosed tumors (group A and a few of group B tumors) can be treated with focal therapy even without chemotherapy, while more advanced tumors need chemotherapy and sometimes radiation and/or enucleation, so the more advanced tumor at diagnosis the more treatment burden for the patient (34, 35). In this series, 25% of the affected eyes among Jordanian patients mandated primary enucleation, while 48% of the affected eyes among refugees mandated primary enucleation, as they presented with a more advanced stage. On the other hand, the chance for eye salvage was almost the same for the eyes that received conservative therapy (80% for Jordanian and 62% for refugees, p=0.06), probably a reflection of the unified treatment protocols in a specialized cancer center with the same team (13). The slight non-significant difference in the outcome might be a reflection of the difficulty of strict follow up for refugees living in camps where transportation and access to the central city for treatment is cumbersome. Eventually, the difference in the mortality rate for both Jordanians and refugees with Rb was not statistically significant (3% for Jordanian patients and 7% for refugees; p=0.33), a potential reflection of the centralization of care for all Rb patients in Jordan (13).

Policymakers should be aware that implementing cancer control programs across the continuum, from early detection to survivorship and palliative care programs, is cost-effective. For example, the 2019 World Health Organization report on cancer found that for every 1 USD invested in cancer, there is a direct productivity return of 2.30 USD and a total social return of 9.50 USD (23, 36). Although this might be less clear for rare diseases like Rb, Despite the clear unwarranted effect of delays in the diagnosis, thus the treatment for Rb patients in terms of decreased eye salvage rate and increased treatment burden, the mortality rates could be maintained at low levels once the patients are diagnosed and treated in specialized centers. An important issue with cancer care is the complicated management protocols, mandating a multidisciplinary-team approach that involves surgery, chemotherapy, radiation therapy, psychosocial support, supportive/palliative care, survivorship, and genetic counseling (13, 29), Therefore, policymakers should be advocates to manage refugees cancer patients in specialized tertiary cancer centers to achieve the best possible survival for this category of patients.

Our study shed light on the problem of the diagnosis and the management of Rb among refugees. We believe that highlighting these problems among refugee patients with rare tumors like Rb will help with developing solutions within healthcare systems that are directed toward refugees mainly in host countries with limited resources (like Jordan). We have shown that even with limited resources, having such rare diseases managed exclusively in specialized referral centers could offer the best outcome to those patients and could save lives. Our study has several limitations that need to be considered. This was a retrospective study with a limited number of patients from a single institution; thus, the findings might not be generalizable to the entire refugee population in Jordan or throughout the region. Nonetheless, the current study has several strengths; the data were obtained from the single referral cancer center (that exclusively treats all patients with Rb in the country) that has previously hosted and currently hosts a large number of refugees.

### 4.1 Strength and limitation

Our study was based on some strengths as all cases were under a strict follow-up program and they were treated in a single tertiary center at KHCC. This is due to that there is an unofficial agreement between Jordanian ophthalmologists that all Rb cases should be referred immediately to where all the cases are treated. On the other hand, It had some limitations as it can be improved by increasing the sample size which could have generated more accurate results, and integrating additional methods of data collection as a questionnaire could have increased the scope and depth of analysis.

## Data availability statement

The raw data supporting the conclusions of this article will be made available by the authors, without undue reservation.

## Ethics statement

The studies involving human participants were reviewed and approved by King Hussein Cancer Center IRB. Written informed consent from the participants’ legal guardian/next of kin was not required to participate in this study in accordance with the national legislation and the institutional requirements. Written informed consent was obtained from the individual(s) for the publication of any identifiable images or data included in this article.

## Author contributions

Conceptualization: YY, QA, MoM, IJ, MuM. Methodology: YY, HH, JK, MS, IS, MA-H, IA-N, MA. Software: RA, OA-H, KS. Validation: YY, IA-N, AM. Formal analysis: YY, QA, MoM, IJ, and MuM. Investigation: HH, JK, MS, IS, MA-H, IA-N, MA. Resources: RA, OA-H, KS. Data curation: IA-N, AM. Writing, original draft preparation: YY, QA,MoM, IJ, RA, OA-H,KS. Writing, review and editing: IA-N, AM, MuM, HH, JK, MS, IS, MA-H, IA-N, MA. Supervision: MA-H, IA-N, MM, AM. All authors contributed to the article and approved the submitted version.
